# Supplementary figures and images for: PCSK9 inhibitor effectively alleviated cognitive dysfunction in a type 2 diabetes mellitus rat model
Source: PeerJ. 2024 Aug 14;12:e17676. doi: 10.7717/peerj.17676 (PMC11330219; doi:10.7717/peerj.17676)

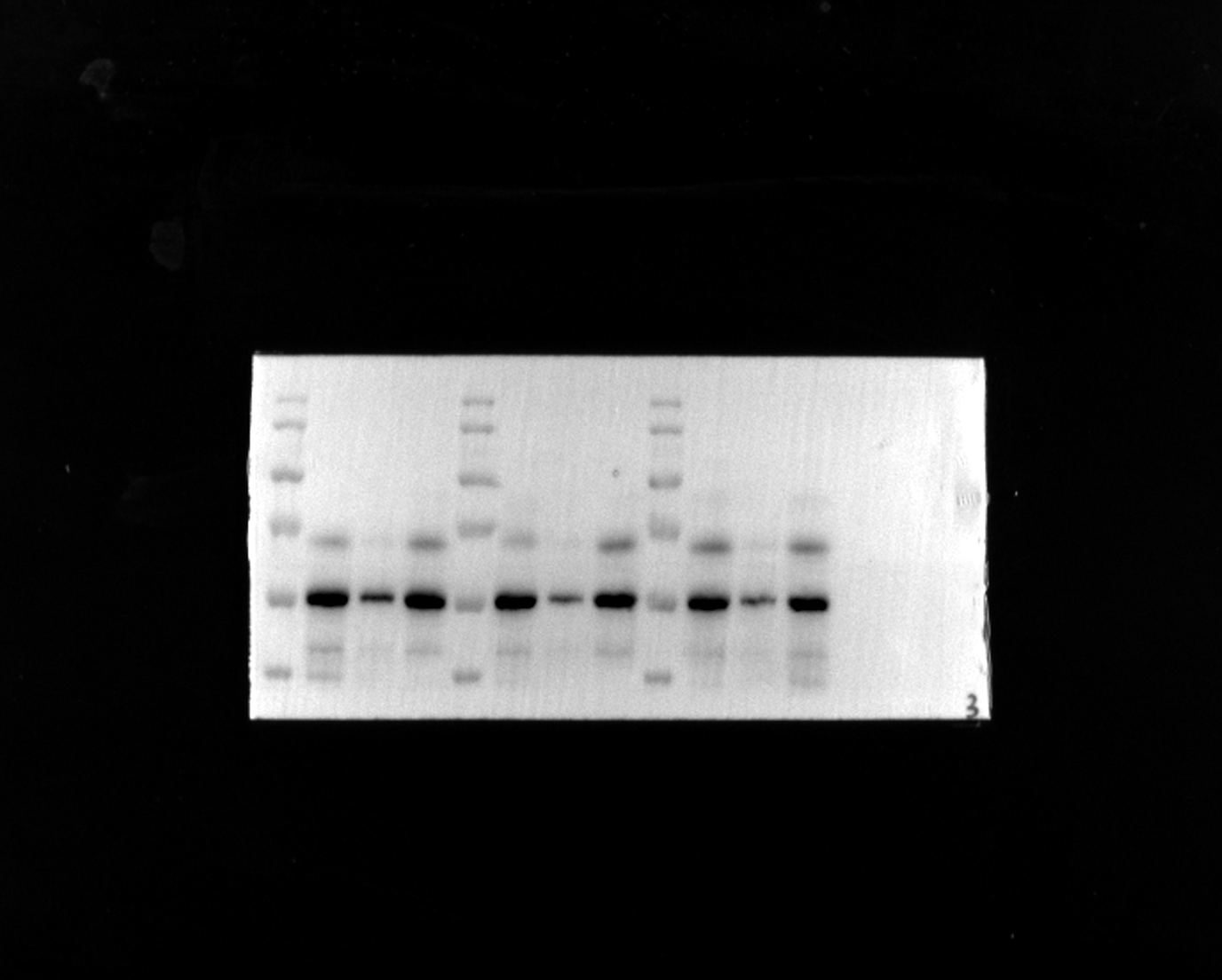

Supplement: Supplemental Information 5 [file peerj-12-17676-s005.zip › western blot/LDL.Tif]

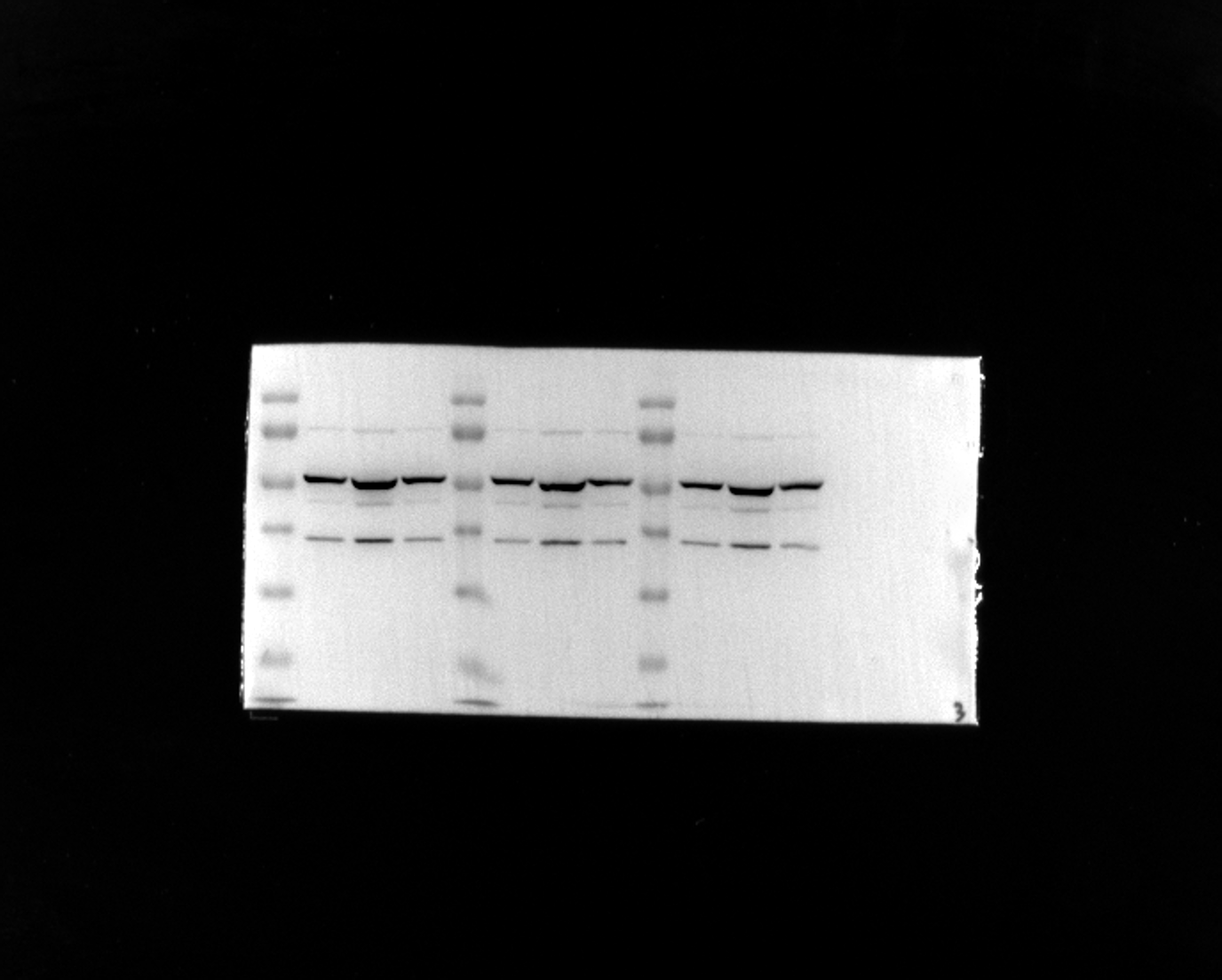

Supplement: Supplemental Information 5 [file peerj-12-17676-s005.zip › western blot/PCSK9.Tif]

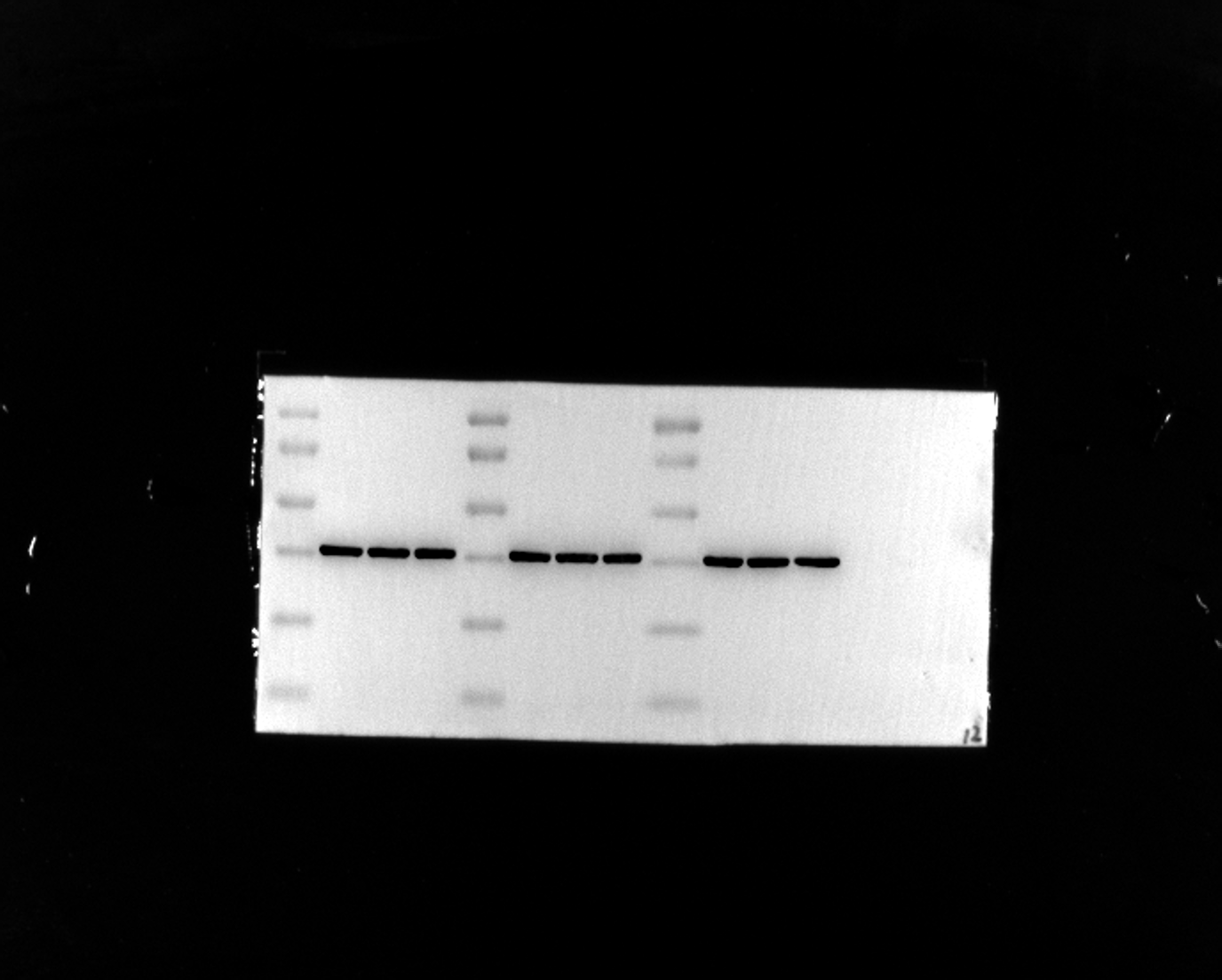

Supplement: Supplemental Information 5 [file peerj-12-17676-s005.zip › western blot/a┬-actin.Tif]
